# Chorea

**Published:** 1846-02

**Authors:** 


*Chorea.*—Dr. Beadle stated that on a former occasion he had reported several cases of chorea, successfully treated by the use of the tincture of black cahash (*actœa racemosa*), that he had recently given it to a patient aged nine years, but not with the usual good effects. He had then resorted to Fowler's solution, five drops three times daily, with the effect of speedly accomplishing a cure.—*Ibid.*

